# Preparedness of the University of KwaZulu‐Natal Occupational Therapy Students and Novice Occupational Therapists for Acute Mental Health Practice: A Qualitative Exploration of Lived Experiences

**DOI:** 10.1155/oti/6484957

**Published:** 2026-07-02

**Authors:** Phakeme Z. Mkhize, December M. Mpanza, Deshini Naidoo

**Affiliations:** ^1^ Discipline of Occupational Therapy, School of Health Sciences, College of Health Sciences, University of KwaZulu-Natal, Durban, South Africa, ukzn.ac.za

**Keywords:** acute mental health, cultural competence, experiential learning, occupational therapy education, South Africa

## Abstract

**Background:**

Occupational therapy students and novice occupational therapists often face considerable challenges when entering acute mental health settings, particularly within South Africa′s culturally diverse and resource‐constrained contexts. This study explored the preparedness of University of KwaZulu‐Natal (UKZN) occupational therapy students and novice practitioners for acute mental health practice within KwaZulu‐Natal′s complex healthcare landscape.

**Methods:**

A qualitative, exploratory design was used with purposive sampling to recruit 15 participants. Ten fourth‐year students and five novice occupational therapists participated in focus group discussions and individual interviews. Data were analysed thematically to identify key patterns related to preparedness, challenges and experiential learning.

**Findings:**

Three overarching themes emerged: (1) educational preparation gaps, (2) practice readiness challenges and (3) systemic and transition constraints. Participants demonstrated strong theoretical knowledge but reported limited practical readiness, particularly regarding culturally responsive care, emotional regulation and evidence‐based intervention in acute settings.

**Conclusions:**

The findings emphasise the need to strengthen occupational therapy education through earlier and more intensive acute mental health exposure, enhanced clinical supervision and greater emphasis on cultural competence and acute care interventions. Grounded in experiential learning theory (ELT), improved experiential learning opportunities may better prepare graduates for effective, contextually relevant practice in South Africa′s acute mental health environments.

## 1. Introduction

Occupational therapy (OT) graduates in South Africa often enter clinical practice with a strong theoretical foundation but limited preparedness for the realities of acute mental health settings [1]. The transition from classroom learning to high‐intensity clinical environments exposes a persistent theory–practice gap, limited exposure to acute psychiatric services and inadequate preparation for culturally responsive practice [2–4]. These gaps raise concerns about whether University of KwaZulu‐Natal (UKZN) OT graduates are adequately equipped to deliver safe, effective and culturally relevant care in KwaZulu‐Natal′s (KZN′s) complex mental health system.

Acute mental health refers to short‐term, high‐intensity psychiatric care for individuals experiencing severe episodes of mental illness that require immediate assessment, stabilisation and therapeutic intervention [5]. Such settings are characterised by rapid patient turnover, unpredictable presentations and complex decision‐making demands. For occupational therapists, acute environments require strong clinical reasoning, quick adaptation of interventions and the ability to build therapeutic rapport within limited time frames [6]. These competencies are critical for supporting recovery and promoting participation in meaningful occupations following acute psychiatric crises. Within mental health care, occupational therapists play a vital role in promoting recovery through engagement in purposeful and meaningful activities. Their practice focuses on assessing the impact of mental illness on daily functioning and developing interventions that enhance coping, emotional regulation and community reintegration [4, 5, 7]. In the South African context, this role becomes more complex due to high rates of trauma, substance use and the social stigma associated with mental illness, requiring practitioners to deliver care that is both evidence‐based and culturally attuned [8, 9].

Internationally, concerns about the preparedness of OT graduates for mental health practice are well documented. A systematic review by Adelle et al. [10] synthesising evidence from Australian OT programmes found that whilst graduates demonstrated adequate theoretical knowledge of mental health frameworks, they consistently reported insufficient practical exposure to acute psychiatric settings, limited training in contemporary intervention modalities and inadequate preparation for the emotional demands of high‐intensity practice. The review highlighted that simulation‐based learning, structured reflective supervision and progressive clinical immersion were associated with improved graduate readiness. Similarly, Steede and Gough [11] examined service user perspectives on OT in acute mental health units and found that therapists were often perceived as underconfident in fast‐paced ward environments, with service users reporting inconsistent therapeutic engagement and limited use of occupation‐focused interventions in acute care. Steede and Gough [11] attributed this partly to inadequate preregistration training for acute contexts, noting that therapists trained predominantly in rehabilitation and community settings struggled to translate their competencies to the distinctly different demands of inpatient acute psychiatry.

Whilst these international concerns are significant, the South African context presents compounding challenges that render the preparedness gap considerably more acute. Unlike Australian or British graduates who practise within relatively well‐resourced and linguistically homogeneous systems, South African occupational therapists must navigate an underfunded public mental health infrastructure, severe human resource shortages and a deeply pluralistic cultural landscape in which biomedical and indigenous healing frameworks coexist and frequently conflict [3, 5, 8, 12, 13]. In KZN specifically, the high burden of trauma, substance use and psychosocial adversity, combined with widespread community stigma and limited access to community‐based mental health services, places exceptional demands on newly qualified practitioners [14, 15]. These contextual realities suggest that the theory–practice gap identified globally is not merely replicated in South Africa but is substantially amplified by systemic inequities and the absence of culturally responsive training frameworks. Addressing graduate preparedness in the South African context, therefore, requires solutions that go beyond curriculum adjustments adopted in high‐income settings, demanding instead a decolonial, context‐sensitive approach that explicitly prepares graduates for the unique social, cultural and resource realities of public‐sector acute mental health practice.

The KZN context further intensifies these challenges. As South Africa′s second‐most populous province, KZN is home to approximately 11.5 million people, with 77.8% speaking isiZulu as their first language [15]. The province′s cultural and linguistic diversity, together with stark inequalities between urban and rural healthcare services, creates complex and multifaceted practice environments [13, 16]. Occupational therapists must navigate diverse cultural understandings of mental illness, language barriers and the coexistence of traditional and biomedical healing systems [3, 14]. Without adequate experiential preparation in such contexts, graduates may struggle to provide care that is both clinically effective and culturally sensitive [17]. At the UKZN, OT students receive mental health training primarily through placements in chronic care facilities from their second to fourth years of study. Whilst students are occasionally placed in specialised psychiatric institutions such as forensic hospitals and selected private clinics, most clinical exposure focuses on long‐term rehabilitation rather than acute care, with the exception of the private facilities [1, 4, 6, 18]. As a result, students have limited opportunities to develop the rapid assessment and intervention skills required in high‐intensity environments. This disconnect reflects the broader theory–practice gap observed in OT education, where conceptual knowledge is not sufficiently reinforced through experiential learning in real‐world contexts [6].

Kolb′s experiential learning theory (ELT) provides a valuable lens for understanding and addressing this educational challenge. According to Kolb, effective learning occurs through a cyclical process involving concrete experience, reflective observation, abstract conceptualisation and active experimentation [19]. In OT education, this experiential cycle allows students to transform theoretical understanding into practical competence through active participation and reflection. However, when clinical experiences in acute settings are limited, students may struggle to engage fully in this learning process, restricting their ability to develop adaptive reasoning, confidence and clinical decision‐making skills required in complex mental health environments.

Given these educational, clinical and contextual challenges, this study explores the preparedness of UKZN OT students and novice graduates for acute mental health practice in KZN. It examines how current undergraduate training, fieldwork exposure and supervisory support at UKZN shape students′ and graduates′ experiential learning processes as described by Kolb [19]. By exploring their lived experiences and perceptions of readiness for practice, the study seeks to generate an insight into how OT education at UKZN influences the competence and confidence of students and graduates in acute mental health contexts. Ultimately, this research contributes to a deeper appreciation of the realities faced by emerging practitioners as they transition from academic learning to professional practice in South Africa′s culturally diverse and resource‐constrained mental health settings.

## 2. Methodology

This study employed a qualitative, exploratory descriptive design to explore the preparedness, challenges and learning experiences of UKZN OT students and novice occupational therapists transitioning to mental health clinical practice. A qualitative exploratory descriptive design enabled an in‐depth understanding of participants′ lived experiences and the contextual factors shaping their preparedness for acute mental health practice, providing insights that cannot be accessed through numerical measurement [20].

### 2.1. Theoretical Framework

This study was underpinned by Kolb′s ELT (1984), which served as the interpretive lens throughout the research process. ELT conceptualises learning as a cyclical process involving four stages: concrete experience, reflective observation, abstract conceptualisation and active experimentation [19]. This framework guided both data collection (interview questions explored participants′ concrete experiences, reflections and application of theoretical knowledge) and data analysis (themes were interpreted through the lens of disruptions or gaps in the experiential learning cycle). The use of ELT was deemed appropriate given the study′s focus on how educational experiences translate into clinical competence in acute mental health practice.

### 2.2. Researcher Positioning and Reflexivity

The primary researcher (P.Z. Mkhize), who conducted all interviews and the focus group, is a female occupational therapist with 9 years′ experience in mental health practice, including work in acute psychiatric settings. At the time of the study, the researcher was a PhD candidate and lecturer in the Discipline of Occupational Therapy at UKZN. The researcher′s dual identity as both educator and clinician, combined with prior professional relationships with some participants (having taught some of the student participants and supervised some clinical placements), positioned the researcher as an insider to the OT community whilst potentially creating power dynamics that could influence data collection and interpretation.

Several measures were implemented to mitigate potential power imbalances and social desirability bias:

1. Student participants were recruited after completion of all academic assessments and grading for their final year, ensuring that participation could not influence their academic outcomes.
2. For novice therapists, only graduates who had completed their studies at least 1–3 years prior were included, removing any direct evaluative relationship.
3. Participants were assured of complete anonymity through the use of pseudonyms and were informed that raw data would only be accessible to the research team.
4. Informed consent emphasised the voluntary nature of participation and the right to withdraw without consequences.
5. Motivated by the research supervisors, the researcher maintained a reflexive journal throughout data collection and analysis, documenting assumptions, emotional responses and potential biases.
6. Regular debriefing sessions with research supervisors (coauthors D. Naidoo and D.M. Mpanza) provided opportunities to challenge interpretations and identify potential researcher bias.

The researcher′s background in acute mental health practice was explicitly disclosed to participants at the start of each interview, with emphasis placed on the researcher′s interest in improving educational outcomes rather than evaluating individual performance. The researcher′s positionality as someone who had navigated similar educational and clinical transitions provided rapport and trust, as evidenced by participants′ willingness to share critical perspectives and vulnerabilities. However, this shared professional identity may have also resulted in certain assumptions being left implicit rather than explicitly explored during interviews.

### 2.3. Participants and Sampling

Participants were recruited through purposive sampling across two groups. The researcher sent email invitations to all fourth‐year OT students who had completed mental health placements in the last fieldwork block after internal and external examinations, followed by an information session explaining the study. Interested students contacted the researcher directly. For novice therapists, recruitment occurred through telephonic recruitment via WhatsApp, where a message for the study was shared by one participant in the alumni–student group, whom the researcher knew and still has contact with. Of 15 fourth‐year students who met inclusion criteria, 10 agreed to participate, three declined due to time constraints, and two did not respond. Of eight novice therapists contacted, five agreed to participate, and three did not respond.

The first group comprised 10 fourth‐year OT students who had completed clinical placements in mental healthcare facilities (King DiniZulu Hospital, Akeso Psychiatric Clinic and Newlands Park Centre). Two of these facilities provided acute care versus chronic care exposure, given the study′s focus on acute mental health practice. Inclusion criteria required the completion of at least one mental health placement in an acute mental health setting or where chances of seeing acute mental health cases would have been provided. The second group included five recently graduated occupational therapists within their first 1–2 years of practice, including community service therapists, all with experience in mental health practice settings. See Table 1 in the Findings section.

**Table 1:** Representative questions for each domain.

#	Domain	Representative question(s)
1	Undergraduate training and curriculum	How would you describe your mental health training during your undergraduate studies? To what extent did your training prepare you for acute mental health practice specifically? What aspects of the curriculum were most useful, and what gaps did you identify?
2	Clinical placement and fieldwork experience	Tell me about your clinical placements in mental health settings. How did your placement experiences prepare you for acute care? What challenges did you encounter, and how did the placement setting shape your learning?
3	Readiness for acute mental health intervention	Describe a situation where you needed to apply occupational therapy skills that you did not learn in class in an acute mental health context. How prepared did you feel to assess, intervene and adapt your approach? Were there specific intervention approaches such as that you felt underprepared to use? Probe: Such as DBT, crisis management or discharge planning?
4	Supervision and reflective learning	How would you describe the quality and consistency of supervision you received during your training and/or early practice? In what ways did supervision help you process your clinical experiences and develop your practice? Were there times when you felt unsupported or that supervision was insufficient? What would you change or recommend be done differently?
5	Cultural and linguistic competence	How did your training prepare you to work with patients from diverse cultural backgrounds? Probe: Particularly where traditional or spiritual beliefs about mental illness were present? How do you navigate situations where a patient′s or family′s cultural understanding differs from the biomedical framework you learned on campus? Were you taught how to work alongside traditional healers or engage with indigenous belief systems? Other spiritual healers?
6	Emotional and psychological preparedness	How did your training prepare you for the emotional and psychological demands of working in acute mental health settings? How do you manage the emotional impact of working with acutely unwell patients, including those presenting with trauma, suicidality or severe psychiatric crises? Were you taught coping skills as a therapist pertaining to protecting yourself and your mental health? Probe: Strategies for self‐regulation, professional boundary setting or preventing burnout?
7	Systemic and contextual factors	Describe the setting in which you completed your mental health placement or currently practise. How do resource availability, staffing levels and the public/private nature of the setting affect your ability to provide occupational therapy? How has exposure, or lack of exposure, to rural or underresourced settings shaped your sense of preparedness?

### 2.4. Data Collection

#### 2.4.1. Setting and Context

Data were collected between December 2023 and June 2024. The focus group with student participants was conducted in a private multipurpose seminar room in the OT department at UKZN′s Westville campus. Individual and dyadic interviews with novice therapists were conducted in private locations chosen by participants, including private meeting rooms at the hospitals where they worked and via a secure Teams meeting due to geographic distance. All settings were chosen to ensure privacy, minimise potential power imbalances and accommodate participant preferences and comfort. No other individuals were present during data collection besides the researcher and participant(s).

#### 2.4.2. Interview Guide Development

A semistructured interview guide was developed based on a review of literature on OT education, preparedness for practice and Kolb′s ELT. The interview guide was pilot‐tested in November 2023 with one recent graduate who conducted clinical supervision in a mental health fieldwork and was not included in the main study. The pilot resulted in minor modifications to simplify language in two questions and add prompts about cultural competence. The same core questions were used across both participant groups; however, the framing was adapted to context: Students were asked about their preparation and anticipated challenges for acute mental health practice, whilst novice occupational therapists were asked about their actual experiences in practice. This allowed for a direct comparison between perceived readiness during training and the realities encountered in early career practice.

The guide was organised around seven domains directly aligned to the study aim of exploring preparedness for acute mental health practice. Representative questions for each domain are provided in Table 1.

#### 2.4.3. Data Collection

Data were collected using semistructured interviews and a focus group to accommodate participant availability and capture both collective and individual perspectives on preparedness for acute mental health practice. A single 60‐min focus group (*n* = 10) was conducted with the student participants to encourage peer interaction and coconstruction of meaning, a valuable approach when exploring shared educational experiences [21].

Three individual interviews and one dyadic interview were conducted with novice occupational therapists to allow flexibility and participant comfort when discussing early career challenges. Individual interviews ranged from 45 to 60 min. The dyadic interview lasted 58 min. The dyadic format was used at the participants′ request due to overlapping schedules and shared work contexts. Individual interviews allowed for a deeper exploration of personal experiences, including the quality of supervision and professional identity. Including both students and novice graduates allowed for a comparison between academic preparation and the realities of early practice.

Data collection continued until saturation was achieved, that is, when no new themes or codes emerged [22]. After completing the focus group and the first two individual interviews, the primary researcher and supervisors reviewed preliminary codes and emerging patterns. As subsequent interviews were conducted, the researcher noted that participants were expressing similar experiences, and no substantially new codes were being generated. After the final interview, the research team reviewed all coded data and confirmed that thematic saturation had been reached, as the last two interviews yielded no new themes and primarily reinforced existing patterns. The single focus group with students was deemed sufficient given that (1) the group format facilitated rich discussion with multiple perspectives represented, (2) themes emerging from student data aligned with and were triangulated by novice therapist data, and (3) students expressed highly consistent views on educational preparation gaps.

All interviews were audio‐recorded with consent using a digital audio recorder with backup recording on a password‐protected smartphone. An assistant researcher was present for the focus group and assisted with brief field notes immediately, documenting contextual observations, nonverbal cues, reflections on interview dynamics and emerging analytical ideas.

### 2.5. Data Analysis

All audio recordings were transcribed verbatim by a research assistant, bilingual in IsiZulu and English. Transcripts were checked for accuracy by the primary researcher by listening to recordings whilst reading transcripts and correcting any errors. Transcripts were managed manually without software. All identifying information was removed from transcripts and replaced with pseudonyms.

Data were analysed using [23]) reflexive thematic analysis, which aligns with the constructivist epistemology underpinning this study ([23]. The analysis was primarily inductive, allowing themes to emerge from the data; however, Kolb′s ELT served as a deductive interpretive lens during later stages of analysis, helping to identify how participants′ experiences reflected specific disruptions in the learning cycle. The coding tree (Table 2) illustrates how initial codes were clustered into subthemes and subsequently consolidated into three overarching themes, which are summarised in Table 3.

**Table 2:** Coding tree—from codes to subthemes to themes.

Codes (initial/focused)	Subtheme	Theme
Limited mental health curriculum time, physical/paediatric bias, unclear OT role in mental health, late introduction to mental health placements and lack of acute‐specific clinical hours	1.1 Theory–practice gap	Theme 1: Educational preparation gaps
Inadequate DBT and crisis management training, superficial CBT coverage, lack of suicidality and trauma response skills, limited occupationally focused crisis strategies and unpreparedness for cultural–spiritual presentations	1.2 Contemporary modalities and crisis skills	
Inconsistent academic vs. clinical supervision, minimal feedback in clinical venues, variable supervisor availability, limited reflective debriefing and preference for clinical over academic supervisors	1.3 Supervision and feedback quality	
Late mental health placement scheduling, limited forensic exposure, insufficient coverage of SIPD and mood disorders, narrow diagnostic range during training and confusion between diagnostic categories	1.4 Curriculum timing and diagnostic exposure	
Discharge planning taught theoretically only, limited practical application, unpreparedness for return‐to‐work assessments and gaps in community reintegration planning	1.5 Discharge planning and reintegration	
Difficulty translating biomedical concepts to isiZulu, navigating ancestral/spiritual explanatory models, limited teaching on cultural safety, unpreparedness for traditional healer collaboration and community stigma navigation	2.1 Cultural and linguistic competence	Theme 2: Practice readiness challenges
Empathy fatigue and vicarious trauma, unpreparedness for emotional boundary setting, limited self‐regulation strategies, countertransference awareness gaps, burnout risk and no formal debriefing structures	2.2 Emotional and psychological preparedness	
Public vs. private resource disparities, understaffing in public hospitals, inadequate MDT collaboration in underresourced settings, absence of rural placement exposure and class‐based differences in family involvement	3.1 Resource and setting disparities	Theme 3: Systemic and transition constraints

**Table 3:** Thematic structure—themes and subthemes.

Theme	Subthemes
Theme 1: Educational preparation gaps	1.1 Theory–practice gap
	1.2 Contemporary modalities and crisis skills
	1.3 Supervision and feedback quality
	1.4 Curriculum timing and diagnostic exposure
	1.5 Discharge planning and reintegration
Theme 2: Practice readiness challenges	2.1 Cultural and linguistic competence
	2.2 Emotional and psychological preparedness
Theme 3: Systemic and transition constraints	3.1 Resource and setting disparities

The six‐phase process proceeded as follows:

1. Familiarisation: The primary researcher checked all transcripts for accuracy and then read and reread the data multiple times whilst making initial notes.
2. Coding: The primary researcher conducted initial line‐by‐line coding of all transcripts. All coding was conducted by the primary researcher, with regular review meetings with supervisors to discuss emerging codes and interpretations. Codes were generated using manual coding over typed and printed transcripts. These were then photocopied for the supervisors during review meetings, capturing semantic and latent content relevant to preparedness, challenges and learning experiences.
3. Theme generation: Codes were collated into preliminary themes based on patterns of meaning across the dataset. The research team met to review preliminary themes and consider how they related to the experiential learning cycle.
4. Theme review: Themes were refined by reviewing coded data extracts to ensure internal coherence and checking themes against the entire dataset to ensure they accurately represented participant experiences.
5. Theme definition: Final themes and subthemes were defined, named and organised to tell a coherent narrative about preparedness for acute mental health practice.
6. Reporting: Themes were interpreted through the lens of Kolb′s ELT, with supporting quotations selected to illustrate key points whilst representing the breadth of participant voices.

### 2.6. Ethical Considerations

Ethical approval was obtained from the Biomedical Research Ethics Committee (BREC/00004840/2022). Participants provided written informed consent after being informed of the study′s purpose, procedures and their rights. Confidentiality was ensured through pseudonyms and secure data storage. Participation was voluntary, with the right to withdraw at any stage. The researcher maintained professional boundaries, ensuring the interviews remained focused on research rather than counselling. Participants showing signs of distress were offered pauses or referrals to appropriate support services.

### 2.7. Trustworthiness

Methodological rigour was ensured using Lincoln and Guba′s [24] criteria of credibility, transferability, dependability and confirmability. Credibility was supported through verbatim transcription. Member checking was not conducted due to the time constraints of participants during their community service year. However, credibility was enhanced through triangulation of student and novice therapist perspectives, supervisory review of interpretations and maintenance of a reflexive journal [25]. Transferability was approached through a rich description of participant experiences rather than through broad generalisation. Dependability was enhanced by maintaining a full audit trail of interview guides, field notes and coding records. Confirmability was strengthened through ongoing reflexivity, with the primary researcher maintaining a reflexive journal and engaging in supervisory discussions to identify and challenge potential biases [26].

## 3. Findings

### 3.1. Participant Characteristics

This study included 15 participants: 10 fourth‐year OT students and five novice occupational therapists. All participants were based in KZN at the time of data collection. The student group comprised seven females and three males, aged 20–25 years, all of whom had completed final‐year mental health clinical placements. The novice therapist group included four females and one male, aged 23–27 years, representing different practice contexts including community service positions in rural district hospitals and urban public hospitals, as well as post–community service positions in urban private clinics. Table 4 provides detailed participant demographics.

**Table 4:** Participant demographics.

Participant ID	Group	Gender	Age range (years)	Current role/year	Current practice setting
S1–S10	Fourth‐year OT student (*n* = 10)	7 F & 3 M	20–25	Final‐year student	N/A
OT A	Novice OT	F	23	Community service (Year 1)	Rural district hospital
OT B	Novice OT	F	24	Post–community service (Year 1)	Urban private clinic
OT C	Novice OT	M	26	Post–community service (Year 2)	Urban public hospital
OT D	Novice OT	F	27	Community service (Year 1)	Urban public hospital
OT E	Novice OT	F	23	Post–community service (Year 1)	Urban private clinics

### 3.2. Overview of Themes

Three overarching themes were identified through reflexive thematic analysis: (1) educational preparation gaps, (2) practice readiness challenges and (3) systemic and transition constraints. These themes are presented visually in the coding tree (Table 3) and collectively illustrate how participants′ learning and transition experiences reflected disruptions within Kolb′s ELT cycle. Each theme is described below with supporting quotations from participants.

## 4. Theme 1: Educational Preparation Gaps

This theme encompasses participants′ perceptions of gaps in their undergraduate education that limited their readiness for acute mental health practice. From an ELT perspective, these gaps reflect insufficient concrete experiences in acute settings and limited opportunities to engage in the full experiential learning cycle during their education. The subthemes below illustrate specific areas where educational preparation did not adequately support the transformation of theoretical knowledge into practical competence.

### 4.1. Theory–Practice Gap

Participants consistently highlighted a strong theoretical base that did not translate into confident, contextually appropriate action in acute mental health settings. This disconnect reflects a breakdown in Kolb′s experiential learning cycle, specifically the inability to move from abstract conceptualisation (classroom learning) to active experimentation and concrete experience (real‐world application). They perceived that curriculum time was heavily weighted towards physical and paediatric practice, leaving limited opportunity for immersive learning in mental health. As a result, students often entered clinical placements uncertain about how to apply theoretical models within the fast‐paced, unpredictable realities of acute psychiatric environments.

My first initial response would be no. Because there isn′t a lot of time focused, I′m talking from a mental health perspective. So obviously now I don′t see any physical patients, I don′t see any paediatric patients, and a lot of the time in varsity was spent on that… I definitely feel that there wasn′t enough time spent or wasn′t enough energy and skills learned in the mental health aspect of things.

—Novice OT A

I didn′t even understand what I was actually doing as an OT, so I wasn′t motivated to be like, I′m an OT, I′m an OT student. I was like, what′s going on here? (mental health institution) What′s going on here? So, I feel like bringing that [acute mental health] exposure a little bit earlier may ignite that something about to some of the students okay, this is what OT is, will they get that?

—Participant B (student group interview)

I wouldn′t say I was adequately prepared enough, it was six weeks, and for the first week I was trying to find my feet because it′s a new setting.

—Participant H (student group interview)

Having the theory, and it′s time to come into the practice, and you′re practising, but then you′re not practising enough so you are not in the right environment to practise, but it′s just not enough, it′s not adequate.

—Participant E (student group interview)

Limited early exposure to acute mental health practice disrupted the experiential learning process. Without progressive engagement throughout the undergraduate curriculum, participants struggled to link abstract concepts from coursework to the concrete experiences and reflective processes needed for clinical reasoning in acute care. This represents a missed opportunity for iterative learning cycles that would allow students to experience, reflect, conceptualise and experiment repeatedly before graduation.

### 4.2. Contemporary Modalities and Crisis Skills

Participants identified inadequate exposure to contemporary and crisis‐relevant OT modalities, such as dialectical behaviour therapy (DBT), de‐escalation techniques and occupationally focused crisis management strategies essential for acute care practice. This gap in the active experimentation phase of Kolb′s cycle meant students had limited opportunity to practice applying contemporary approaches in supervised settings before entering independent practice. Although cognitive behavioural therapy (CBT) was introduced during training, students felt underprepared to employ newer, contextually relevant approaches used in modern psychiatric services.

I think we were taught a lot of CBT. And I think now that I′m predominantly working in a DBT environment, maybe just needing to cover that a bit and how the DBT principles work and things like that, because it was gone over vaguely, but I don′t think in enough depth.

—Novice OT A

Also something we′ve learned recently is using the OT grow model, which is very patient led, instead of therapist led, we are just guiding them, but their reflections are coming from their experiences.

—Novice OT D

No, we didn′t learn how to handle a patient who′s just experienced something traumatic or someone who reports being suicidal. That could also be a good addition because yeah, it is relevant to our context as well.

—Novice OT A

Participants also highlighted a lack of preparation for working with clients who present with culturally embedded understandings of mental illness.

Balancing spirituality or traditional calling and drawing the line between that and mental illness wasn′t directly taught to us…and that′s also about cultural inclusivity as well and cultural awareness.

—Novice OT A

In campus, the focus was more psychosis and substance‐induced… but working here I see people with daily life issues coming all the way back from their childhood experiences, attachment, and trauma. What we missed out on in university was the link between emotions, behaviour, and actions, and more focus on daily coping strategies.

—Novice OT E

It is something that actually bothered me for some time, even in my third year, I didn′t feel adequately prepared, and even now I′m not confident when it comes to reintegrating, especially when we′re talking about returning to work.

—Novice OT D

Without practice in applying DBT principles, crisis intervention strategies or cultural negotiation techniques during training, graduates entered practice unprepared for the modalities and situations they would encounter.

### 4.3. Supervision and Feedback Quality

Supervision emerged as a critical factor influencing learning quality. From an ELT perspective, supervision facilitates the reflective observation phase by providing structured opportunities for students to process experiences, receive feedback and refine their understanding. Participants described variability in both academic and clinical supervision, with some reporting minimal guidance in clinical settings, whilst others experienced supportive, collaborative supervision.

I think in terms of the university, I think the supervision that we were receiving was adequate… but it was in much contrast with the actual supervision in the venue. It was like, I feel like in the venue, it was more just like, you guys just do your thing, like there was very little supervision.

—Participant B (student group interview)

I feel like both of them [supervisors] gave us. One [academic supervisor] gave us the 50%, and the other one [clinical supervisor] was giving us the other 50% in a way.

—Participant G (student group interview)

At that facility… there were three full‐time OTs, so they were very involved… I found I was more open to asking my clinical supervisor for feedback than the academic supervisor.

—Participant I (student group interview)

The feedback I received from the clinical supervisor made such a huge difference, even though in my first week I didn′t know what was going on, as I practised more it was the feedback that helped me see the difference.

—Participant J (student group interview)

In terms of my academic supervisor, there was a lack of empathy and understanding for me, it was very didactic, do this, do that. There wasn′t too much emotion to it for me to feel like this person understands where I′m coming from.

—Participant E (student group interview)

Inconsistent supervision disrupted reflective observation and limited students′ opportunities for constructive feedback, a core element in experiential learning. Without regular, high‐quality supervision, students could not adequately process their concrete experiences or receive guidance on how to improve their practice.

### 4.4. Curriculum Timing and Diagnostic Exposure

Participants endorsed the need for earlier and more progressive exposure to mental health settings. Delayed introduction to mental health placements restricted opportunities for iterative experiential learning cycles. Limited diagnostic diversity, particularly in forensic psychiatry and substance‐induced psychotic disorders, left graduates uncertain in these environments.

I sort of do not have any training when it comes to forensics. So I just don′t really understand what to do there… But as an OT, you know better than to just follow a mere referral, you need to dig deeper.

—Novice OT B

When we did psych in varsity, it was a lot of the more psychotic acute conditions… but here it′s more the depression and anxiety… it was gone over very quickly.

—Novice OT A

I′m seeing a lot of substance‐induced psychotic disorder (SIPD) cases. SIPD was not really thoroughly covered… I even had a question about the differentiation between SIPD and schizophrenia.

—Novice OT B

When it came to working with high‐functioning clients with seasonal affective disorder, which I only came to know about this year, and SIPD, I had to find a way to navigate care for those patients because my third year exposure was not diverse.

—Novice OT D

What we missed out on in university was more focus on daily coping strategies, it was more psychosis and substance‐related that we saw there, but over here it′s a lot of daily life issues, attachment, and trauma.

—Novice OT E

The narrow range of diagnostic presentations encountered during training limited the practitioners′ concrete experiences and reduced their confidence in managing the full spectrum of mental health conditions encountered in acute settings.

### 4.5. Discharge Planning and Reintegration

Although discharge planning was well covered theoretically, practitioners lacked practical opportunities to apply it in real cases. This represents a gap between abstract conceptualisation (theoretical knowledge) and active experimentation (practical application) in Kolb′s learning cycle. This gap affected their readiness for patient reintegration, particularly regarding return‐to‐work assessments.

In theory, discharge planning was always emphasised… but in practical, we didn′t get much of a chance to do it.

—Novice OT B

I didn′t feel adequately prepared… especially when we′re talking about returning to work.

—Novice OT B

These findings illustrate how curriculum design limited the integration of abstract conceptualisation with concrete experience, sustaining the theory–practice divide and leaving graduates unprepared for critical components of acute care delivery.

## 5. Theme 2: Practice Readiness Challenges

This theme addresses participants′ readiness to manage the cultural, linguistic and emotional complexities of acute mental health practice. These challenges reflect limitations in both the concrete experience phase (insufficient exposure to diverse cultural contexts) and the reflective observation phase (limited opportunity to process emotional responses) of the experiential learning cycle.

### 5.1. Cultural and Linguistic Competence

Cultural and linguistic diversity presented major challenges in clinical communication, assessment and intervention. Students reported difficulty translating biomedical concepts into indigenous languages and struggled to explain psychiatric conditions in culturally sensitive ways. This represents a gap in concrete experiences with cultural negotiation and insufficient reflective processing of how to bridge biomedical and indigenous understandings of mental illness.

You have to explain to gogo [grandmother] that she′s not a witch… you are approaching a culture where a person running naked at night is seen as bewitched.

—Participant E (student group interview)

As a Zulu‐speaking person, it was a challenge to translate the theory that I learned… I wish it could be culturally sensitive and culturally appropriate. I think that′s where the university failed me.

—Participant E (student group interview)

Participants also emphasised the need to address community stigma and culturally rooted misconceptions about mental illness.

They believe you′ve been bewitched, or my ancestors are angry… it′s never substance use or trauma. It′s always sangoma [spiritual healer] or ancestors.

—Novice OT C

We should be taught ways on how to handle those type of things [community stigma and beliefs] other than advocating because advocating is not enough.

—Participant G (student group interview)

With some theoretical tools, like psychodrama or psychotherapy, you experience challenges when trying to apply them to maybe a black man, the theory does not speak to the culture of your clients or their background.

—Novice OT D (individual interview)

This theme reflects a breakdown in experiential exposure to real cultural interactions that foster transformative learning, a key component of Kolb′s reflective observation. Without structured opportunities to engage with diverse cultural belief systems during training and without guided reflection on how to navigate these complexities, graduates felt unprepared for the cultural realities of South African mental health practice.

### 5.2. Emotional and Psychological Preparedness

Participants described emotional strain and insufficient training in self‐regulation, resilience and emotional boundary setting, skills critical for sustaining professional wellbeing in high‐intensity environments like acute mental health practice. The lack of structured reflective spaces in training represents a critical gap in the reflective observation phase of ELT, where learners process emotional experiences and develop coping strategies.

Empathy is a big one… but it can also be a negative thing when you′re taking on everyone else′s burdens.

—Novice OT A

These patients are acutely ill… there will be a lot of emotions required from you that you need to learn to control so you′re not showing visible fear or any countertransference.

—Novice OT C

I do not think I was taught on how to block myself out and protect myself from that [severe trauma]. Only now do I know how to deal with that.

—Novice OT B

Many patients with anxiety and depression can make you find yourself operating in that same space… When you go home, you begin to see certain patterns in you that they displayed during the day.

—Novice OT D

Being overly empathetic, absorbing all of my patients′ problems and almost grieving with them initially, until I learned that if I′m always going to place myself in their shoes, I′m not going to be able to show them another path that they need to reach.

—Novice OT E

Most important for me is to be emotionally intelligent and available, because if I am dysregulated and cannot handle my own emotions, it is going to show up in the patients and in the way I handle their caseloads.

—Novice OT C (dyadic interview)

Without structured reflective spaces during training, participants lacked opportunities for emotional processing, a vital element of the reflective observation phase in ELT. This gap left graduates vulnerable to vicarious trauma and burnout, unprepared to manage the psychological demands of acute mental health work.

## 6. Theme 3: Systemic and Transition Constraints

This theme highlights how structural and systemic factors in the healthcare system and educational programme constrained experiential learning opportunities and professional development. These constraints affected all phases of the ELT cycle by limiting the quality and diversity of concrete experiences available during training.

### 6.1. Resource and Setting Disparities

Participants described significant disparities between private and public healthcare settings, which shaped both their learning and professional adjustment. Resource‐rich private settings facilitated interprofessional collaboration, whilst underresourced public hospitals constrained service delivery and learning opportunities. These disparities meant that students′ concrete experiences varied dramatically depending on placement setting, creating unequal preparation for practice.

There are lots more human resources and personal MDT interactions available this side [private facility]… with the government, it was always lots of doctors dealing with different patients at the same time.

—Novice OT D

The main thing that′s preventing us from providing our services… is the lack of resources.

—Novice OT C

There was not enough staff for the amount of patients they had… it was just myself and an OT technician in the psychiatric part of the public hospital.

—Novice OT A

At private hospitals, families are involved and supportive… because of the class that they come from.

—Participant G (student group interview)

If maybe we were given more exposure to rural settings in our practical′s… in psych, I feel that there was a lack of exposure to those settings.

—Novice OT B

In government, because of the lack of resources, you have a certain goal to meet for the day and that doesn′t always give you the time to provide the quality of care that they need, it′s mostly just the priority of what they need.

—Novice OT E

I know of colleagues who didn′t get a fair exposure, they were back‐to‐back on just government, just the same SUD facilities, and that impacted how prepared they felt.

—Novice OT A

These disparities constrained opportunities for authentic experiential learning across diverse practice contexts, reinforcing inequities in clinical preparedness and professional confidence. Students placed primarily in well‐resourced settings gained concrete experiences that did not prepare them for the resource constraints of public sector practice, whilst those in underresourced settings may have had limited exposure to interprofessional collaboration and evidence‐based interventions.

## 7. Discussion

This study provides insights into a multifaceted gap between OT education and the realities of acute mental health practice, as experienced by UKZN students and novice practitioners in KZN. Although participants reported a solid theoretical foundation, three interrelated areas of concern, namely educational preparation gaps, practice readiness challenges and systemic and transition constraints, collectively limit graduates′ ability to deliver effective, contextually responsive services within KZN′s diverse healthcare landscape.

The findings highlight a persistent theory–practice divide and the delayed introduction of mental health fieldwork in the curriculum. According to Kolb′s ELT, deep and adaptive learning occurs through a recurring cycle of concrete experience, reflective observation, abstract conceptualisation and active experimentation [19]. When mental health placements are concentrated in the final year, opportunities for this iterative process are constrained. Students consequently struggle to transform theoretical understanding into professional competence, particularly in unpredictable acute psychiatric settings. Furthermore, limited diagnostic diversity, such as minimal exposure to forensic psychiatry, high‐functioning mood disorders and substance‐induced psychosis, narrows students′ experiential base and undermines their confidence when managing the complexity of acute care. Inadequate training in contemporary approaches, including DBT and crisis intervention strategies, further reduces graduates′ readiness to manage escalating clinical situations. Consistent with best‐practice literature, structured supervision and timely feedback are essential for consolidating competence and professional identity; however, participants described supervision as inconsistent and often fragmented across academic and clinical sites.

The second theme emphasised the cultural, linguistic and emotional demands of acute mental health practice. Students and novice therapists frequently encountered patients whose beliefs were grounded in spirituality, ancestral causation and indigenous health systems. Many struggled to navigate these perspectives within a Western biomedical framework, exposing significant gaps in cultural competence. Campinha‐Bacote′s [27] model conceptualises cultural competence as an ongoing developmental process involving cultural awareness, knowledge, skill, encounters and desire. Viewed through this lens, single lectures or cultural sessions are insufficient for building genuine competence. Similarly, Ramugondo′s [3] decolonial OT framework calls for the integration of indigenous knowledge systems and critical reflection on Eurocentric practice assumptions to promote culturally relevant care. These findings resonate with, yet also extend, the broader international literature on culturally responsive OT education. A recent scoping review of culturally responsive care in OT education and practice found that most published curriculum interventions remain confined to isolated awareness‐raising modules, with critical frameworks such as indigenous epistemologies and anticolonial perspectives still underrepresented ([28]. Comparable gaps are reported in settler colonial contexts such as Canada, where the majority of occupational therapists remain unaware of the traditional healing practices used within their own healthcare regions and report low confidence in supporting clients to access them [29]. What distinguishes the KZN context, however, is the extent to which traditional healing functions not as a peripheral consideration but as a primary explanatory framework for the majority of service users ([30], reflected in participants′ accounts of clients attributing psychiatric symptoms to ancestral displeasure or bewitchment rather than to substance use or trauma. This positions traditional healers not merely as a topic for cultural awareness training but as parallel practitioners with whom occupational therapists already share clients, whether or not this is formally acknowledged [31].

Translating this into curriculum reform requires moving beyond the single lecture or isolated cultural session that participants in this study and comparable South African research described as insufficient for sustained competence [32]. A more concrete approach would embed structured, longitudinal content on indigenous explanatory models of mental illness across multiple years of training, rather than concentrating it within a single preplacement session. This content should be codeveloped, and where possible cotaught, with traditional health practitioners (izinyanga, sangomas and faith healers), drawing on precedents elsewhere in South African health sciences education, where simulated clinical encounters with patients using traditional medicine have been used to build factual knowledge and communication skills whilst explicitly addressing the negative ‘hidden curriculum’ that students otherwise absorb from clinical role models [32]. For OT specifically, this could extend to structured opportunities for students to engage with traditional healers during placements, framing them as part of the client′s occupational and social environment rather than as competitors to biomedical care [30, 31]. Second, the cultural dissonance described by participants, such as the need to reassure a ‘gogo’ that she is not bewitched or to translate biomedical diagnoses into isiZulu without equivalent terminology, points to a need for structured, supervised debriefing sessions immediately following such encounters. Embedding these within the reflective observation phase of Kolb′s cycle would allow students to process the emotional and professional identity dimensions of cultural dissonance as they arise, rather than carrying them unprocessed into independent practice. Establishing peer consultation groups or communities of practice that connect early‐career therapists with more experienced clinicians and, where feasible, with traditional health practitioners would provide an ongoing structure for processing cultural dissonance during the early‐career period when participants in this study reported feeling most unsupported. Third, students would benefit from explicit teaching on existing models of collaboration between biomedical and traditional practitioners in South Africa, ranging from informal mutual referral to more formalised cooperation [33], so that graduates have a working understanding of how to communicate respectfully with clients consulting both systems simultaneously and how to represent these dual treatment pathways within multidisciplinary team discussions. Collectively, these measures would shift cultural competence training from an abstract, awareness‐based exercise towards a concrete, experiential and reciprocal engagement with the belief systems that shape care‐seeking for the majority of clients encountered in KZN′s acute mental health settings.

Equally salient were deficits in emotional and psychological preparedness. Participants described feeling overwhelmed by clients′ trauma and emotional intensity, with limited strategies for self‐regulation, boundary setting or reflective processing. This supports prior research highlighting the importance of emotional intelligence and resilience frameworks in professional training [34]. Embedding structured reflection and peer debriefing into fieldwork could strengthen emotional competence and align with the reflective observation and active experimentation phases of Kolb′s learning cycle.

Finally, the systemic and transition constraints reported by participants, ranging from unequal resource distribution across public and private facilities to limited rural exposure and weak discharge planning training, reflect structural inequities within South Africa′s mental health system [8]. These barriers mirror national concerns about uneven mental health service delivery and human resource distribution [8, 35, 36]. Importantly, they demonstrate that curriculum reform alone is insufficient; rather, a multilevel response involving stronger academic–clinical partnerships, enhanced supervision structures and equitable placement access is required to support experiential continuity and professional growth.

## 8. Study Implications

Addressing these gaps requires a multilayered educational strategy. First, mental health placements should be introduced from the first year onward, with progressively complex rotations across acute and chronic settings to allow repeated experiential learning cycles. Second, supervision protocols must be standardised to ensure timely, constructive feedback and parity across placement sites. Third, cultural competence and decolonial practice modules, codesigned with community stakeholders, should be embedded throughout the curriculum to strengthen students′ ability to work within indigenous belief systems. Fourth, resilience training, emotional regulation and crisis intervention content should be integrated to reduce burnout risk and improve service delivery. Fifth and final, targeted exposure to diverse diagnoses, including forensic psychiatry, high‐functioning mood disorders and substance‐induced psychosis, along with simulation of rural, resource‐limited practice environments, will better prepare graduates for the breadth of South African mental health contexts.

## 9. Conclusion

The findings emphasise the need to strengthen OT education through earlier and more intensive acute mental health exposure, enhanced clinical supervision and greater emphasis on cultural competence and acute care interventions. Improved experiential learning opportunities may better prepare graduates for effective, contextually relevant practice in South Africa′s acute mental health environments. Addressing these deficits through a context‐responsive and decolonial‐informed curriculum, one that integrates early and progressive clinical exposure, structured supervision, cultural competence training and evidence‐based practices, is essential to producing graduates who can deliver safe, effective and culturally attuned mental health services in South Africa.

## 10. Study Limitations

The study was conducted at a single institution with a relatively small, purposively selected sample; therefore, findings reflect the experiences of UKZN graduates and may not represent experiences of graduates from other institutions with different curriculum structures, clinical partnerships or educational approaches. Whilst the geographic focus on KZN provided rich contextual insights, findings may not reflect experiences in other South African provinces with different cultural compositions, resource availability or healthcare infrastructure. Additionally, the study′s focus on acute mental health practice may not reflect preparedness for other mental health service delivery models. The study′s findings may also be influenced by the specific characteristics of the practice settings where participants completed their community service or early career positions. Future research could benefit from multi‐institutional studies comparing graduate preparedness across different educational programmes and longitudinal studies tracking graduate development over extended periods. Additionally, investigation of specific educational interventions designed to address identified gaps and exploration of perspectives from clinical supervisors, patients and other healthcare team members would provide additional insights into graduate preparedness and educational effectiveness.

## Author Contributions

Phakeme Z. Mkhize was the primary researcher responsible for the conceptualisation of the study, data collection, data analysis and writing of the manuscript. Deshini Naidoo and December M. Mpanza were supervisors on the project who contributed to the conceptualisation of the study, data analysis, consultations and critical review of the manuscript.

## Funding

This study was supported by the National Research Foundation (NRF) of South Africa – Black Academics Advancement Program, BAAP2205036083.

## Conflicts of Interest

The authors declare no conflicts of interest.

## Data Availability

The data that support the findings of this study are available from the corresponding author upon reasonable request.
